# Radiosurgery for brainstem metastases with and without whole brain radiotherapy: clinical series and literature review

**DOI:** 10.1007/s13566-016-0281-4

**Published:** 2016-10-27

**Authors:** Louise Murray, Cynthia Menard, Gelareh Zadeh, Karolyn Au, Mark Bernstein, Barbara-Ann Millar, Normand Laperriere, Caroline Chung

**Affiliations:** 1 Department of Radiation Oncology, Princess Margaret Cancer Centre/University of Toronto, 610 University Avenue, Toronto, ON M5G 2M9 Canada; 2 Division of Neurosurgery, University of Toronto, Toronto Western Hospital, Toronto, ON Canada

**Keywords:** Stereotactic radiosurgery, Brainstem metastases, Brain metastases, Whole brain radiotherapy

## Abstract

**Objective:**

The objective of this study was to investigate outcomes for patients with brainstem metastases treated with stereotactic radiosurgery (SRS).

**Methods:**

Patients with brainstem metastases treated with SRS between April 2006 and June 2012 were identified from a prospective database. Patient and treatment-related factors were recorded. Kaplan-Meier analysis was used to calculate survival and freedom from local and distant brain progression. Univariate and multivariate Cox regression was used to identify factors important for overall survival.

**Results:**

In total, 44 patients received SRS for 48 brainstem metastases of whom 33 (75 %) also received whole brain radiotherapy (WBRT): 23 patients (52 %) WBRT prior to SRS, 6 (13.6 %) WBRT concurrently with SRS and 4 (9.0 %) WBRT after SRS. Eight patients received a second course of WBRT at further progression. Median target volume was 1.33 cc (range 0.04–12.17) and median prescribed marginal dose was 15 Gy (range 10–22). There were four cases of local failure, and 6-month and 1-year freedom from local failure was 84.6 and 76.9 %, respectively. Median overall survival (OS) was 5.4 months. There were four cases of radionecrosis, 2 (4.8 %) of which were symptomatic. The absence of external beam brain radiotherapy (predominantly WBRT) showed a trend towards improved OS on univariate analysis. Neither local nor distant brain failure significantly impacted OS.

**Conclusion:**

This retrospective series of patients treated with SRS for brainstem metastases, largely in combination with at least one course of WBRT, demonstrates that this approach is safe and results in good local control. In this cohort, no variables significantly impacted OS, including intracranial control.

**Electronic supplementary material:**

The online version of this article (doi:10.1007/s13566-016-0281-4) contains supplementary material, which is available to authorized users.

## Introduction

Between 20 and 40 % of patients with cancer develop brain metastases of which 2–5 % arise in the brainstem [1–5]. While brainstem metastases represent a small proportion of brain metastases overall, these lesions are challenging in terms of management, and prognosis is traditionally poor. Given the critical functions performed by the brainstem, surgery is not typically utilised, and the preferred treatment is with radiation, either with conventionally fractionated external beam radiotherapy or stereotactic radiosurgery (SRS).

Radiosurgery is an established treatment modality for brain metastases, commonly offered when the metastases are limited in number and size (generally <3 cm in diameter) [6]. The highly conformal nature of SRS treatment allows a much higher dose to be delivered to the tumour than can be achieved with conventionally fractionated radiotherapy, without excessive damage to the surrounding normal tissues.

Since the brainstem is considered an eloquent area, where salvage surgical resection in the event of brainstem radionecrosis is not easily achieved, there have been concerns about the safety of SRS in this location. Given this, and the fact that brainstem metastases are relatively infrequent, the use of SRS in the brainstem is relatively under-represented in the literature. Here, we present the clinical outcomes for 44 patients with 48 brainstem metastases treated with SRS with and without whole brain radiotherapy along with investigation of patient and treatment-related factors that could potentially impact outcomes.

## Methods

### Patients

Following approval by the institutional research ethics board for the period April 2006 to June 2012, patients who received SRS at University Health Network (Princess Margaret Cancer Centre and Toronto Western Hospital) for brainstem metastases during the above time period were identified from a prospective brain metastases database. The following details were recorded: age, gender, primary tumour histology, number of brainstem lesions, brainstem location, presence of additional non-brainstem brain metastases at time of brainstem SRS, extra-cranial disease control at time of brainstem SRS, use of additional radiotherapy and dose, site and timing of additional brain radiotherapy.

### Stereotactic radiosurgery

All patients were treated using the Gamma Knife® 4C or Perfexion™ radiosurgery unit (Elekta AB, Stockholm, Sweden). Patients were immobilised using a Leskell stereotactic frame. Using the GammaPlan® system (Elekta AB, Stockholm, Sweden), patient MR (gadolinium-enhanced T1-weighted and T2-weighted) and CT images were imported for target and organ-at-risk delineation, and radiosurgery plans were generated. All patients received single fraction SRS. The dose prescriptions were based on volume and generally followed the RTOG guidelines, while limiting dose to the surrounding brainstem to try to keep the maximum dose to 1mm^3^ brainstem less than 15 Gy. It was aimed to keep the conformality index less than 2. The following dosimetric parameters were recorded: target volume, largest extent of target, conformality index, gradient index, minimum target dose, maximum target dose, mean target dose, and prescription isodose.

### Patient follow-up

All patients were reviewed prior to SRS in a dedicated multi-disciplinary brain metastases clinic. Following SRS, all patients were followed up in the same clinic at 3-month intervals, with serial brain MRI and neurological assessment. Changes in target lesions were defined according to RECIST criteria [7], as a number of these brainstem metastases did not all meet the minimum diameter requirement of 10 mm described in the Response Assessment in Neuro-Oncology criteria for brain metastases [8].

### Statistics

All statistical analysis was completed using SPSS version 21 (IBM, USA), and *p* < 0.05 was considered statistically significant. The Kaplan-Meier method was used to calculate survival outcomes. Overall survival was defined as the date of Gamma Knife treatment to date of death or censored at last follow-up. For overall survival and distant brain failure, calculations were on a per patient basis (i.e. patients with more than one brainstem lesion were only considered once in the analysis), and where patients had brainstem lesions treated sequentially, overall survival was considered from the date of the first brainstem radiosurgery treatment. Local failure was defined on an individual lesion basis as the date of Gamma Knife treatment to the date of first reported radiological progression. Lesions that progressed on imaging and/or caused new neurologic symptoms but which resolved without any further anti-cancer treatment were considered radionecrosis rather than local progression. Distant brain failure was defined as the development of new metastases in the brain but not within the brainstem.

Univariate and multivariable Cox regression were used to investigate factors predictive of overall survival. For univariate analysis, patient age, gender, primary tumour histology (breast, non-small cell lung vs. other), the use of additional brain radiotherapy, the presence of synchronous non-brainstem brain metastases at the time of brainstem SRS, extra-cranial disease control, local failure and distant brain failure were evaluated. Any covariates with a *p* value of 0.1 or less were intended for inclusion in the multivariable model for overall survival. Factors potentially predictive of local control or radionecrosis were not investigated in a univariate or multivariable context given the low number of events and small sample size.

## Results

### Patients

In total, 44 patients received SRS for 48 brainstem lesions, including 3 patients in whom two brainstem lesions were treated synchronously and one patient who received two sequential SRS treatments to the same brainstem metastasis. Baseline characteristics are shown in Table 1. Median follow-up was 16.1 months (range 0–54.0). Median age at SRS was 58 years (range 32–76) and 36 % of patients were male (*n* = 16). All patients had KPS > 60 at the time of brainstem SRS. The most frequent primary tumour histologies were non-small cell lung cancer (*n* = 14, 32 %) and breast cancer (*n* = 12, 27 %). Brainstem metastases represented the only intra-cranial metastasis in 17 patients (39 %), while 11 patients (25 %) had one other brain lesion at the time of brainstem SRS, and the remainder had two or more additional lesions (up to a maximum of 9 non-brainstem brain metastases). The majority (*n* = 33, 75 %) of patients received whole brain radiotherapy (WBRT) during their course of care: 23 patients (52 %) had received WBRT prior to SRS to the brainstem metastases, 6 patients (13.6 %) received WBRT concurrently with brainstem SRS (defined as within 4 weeks of SRS) and 4 patients (9.0 %) received WBRT after SRS. A further two patients received base of skull radiotherapy prior to brainstem SRS and one additional patient received radiotherapy for a temporal bone lesion prior to brainstem SRS. In 8 patients, a second course of WBRT was also delivered at further progression, and in 1 patient, who received base of skull radiotherapy prior to brainstem SRS, WBRT was delivered at further progression.

**Table 1:** Baseline characteristics (no. patients: 44, no. lesions: 48)

Factor	*n*	%
Age	Median 58	Range 32–76
Gender		
Male	16	36.4
Female	28	64.6
Primary tumour:		
Breast	12	27.3
Non-small cell lung cancer	14	31.8
Gynaecological	6	13.6
Melanoma	3	6.8
Renal	2	4.5
Colorectal	2	4.5
Other	5	11.4
Site of brainstem metastasis		
Midbrain	5	10.4
Pons	29	60.4
Medulla	6	12.5
Pons-midbrain	3	6.3
Pons-medulla	5	10.4
Number of brainstem lesions		
1	40	90.9
2 treated synchronously	3	6.8
2 treated sequentially	1	2.3
Additional non-brainstem brain metastases at time of brainstem SRS		
0	17	38.6
1	12	27.3
2	2	4.5
3	7	15.9
>3	6	13.6
Other brain radiotherapy		
None	8	18.2
WBRT prior to SRS	23	52.3
Other brain RT prior to SRS^a^	3	6.8
WBRT concurrent with SRS	6	13.6
WBRT after SRS	4	9.1
Re-WBRT after SRS	8	18.2
Extra-cranial disease status at time of SRS		
Controlled	20	45.5
Uncontrolled	18	40.9
Unknown	6	13.6

*SRS* stereotactic radiosurgery, *WBRT* whole brain radiotherapy

^a^Two patients have base of skull radiotherapy and one had IMRT for a temporal bone lesion

### Dosimetry

Dosimetric factors are summarised in Table 2. The most commonly prescribed marginal dose was 15 Gy (*n* = 38 lesions, 79.2 %), most often prescribed to the 50 % isodose (median prescription isodose: 50 %, range 39–80 %), resulting in a median mean dose of 20.8 Gy (range 13.9–35.0). The median brainstem metastasis volume was 1.33 cc (range 0.04–12.17 cc). Plans were highly conformal with steep dose gradients (Table 2), thus limiting the dose to the surrounding normal tissues. Of the 33 patients who received WBRT, 18 (54.5 %) received 20 Gy in 5 fractions, 14 (31.8 %) received 30 Gy in 10 fractions and 1 (2.3 %) received 25 Gy in 10 fractions. Both of the patients who received base of skull radiotherapy prior to SRS received 20 Gy in 5 fractions and the one patient who had received radiotherapy for a temporal bone lesion received 55 Gy in 50 fractions in a BID schedule resulting in a maximum brainstem dose of 22 Gy. Of the 8 patients who received 2 courses of WBRT, patients largely received 20 Gy in 5 fractions for their first course of WBRT, with just 2 patients who received 30 Gy in 10 fractions. Half of patients received 25 Gy in 10 fractions for their second course of WBRT, although 20 Gy in 10 fractions and 15 Gy in 5 fractions were also delivered as the second course. The full summary is provided in Online Resource 1. One patient who had received skull base radiotherapy, 20 Gy in 5 fractions, prior to brainstem SRS also went on to receive WBRT following brainstem SRS at a dose of 25 Gy in 10 fractions.

**Table 2:** Dosimetric parameters

Parameter	Median	Range
Target volume (cc)	1.33	0.04–12.17
Largest extent of target (mm)	16.95	4.70–37.00
Prescribed dose (Gy)	*n*	%
15 Gy	38	79.2
12 Gy^a^	6	12.5
10 Gy	1	2.1
14 Gy	1	2.1
18 Gy	1	2.1
22 Gy	1	2.1
Prescription isodose (%)	50	39–80
Conformality index	1.25	1.04–2.85
Gradient index	2.90	2.29–4.44
Target minimum dose (Gy)	12.90	7.94–19.56
Target maximum dose (Gy)	29.94	18.76–48.98
Target mean dose (Gy)	20.83	13.89–34.96

^a^This dose was used for the one case of SRS re-treatment of a brainstem metastasis

### Survival and brain response

**Fig. 1:**
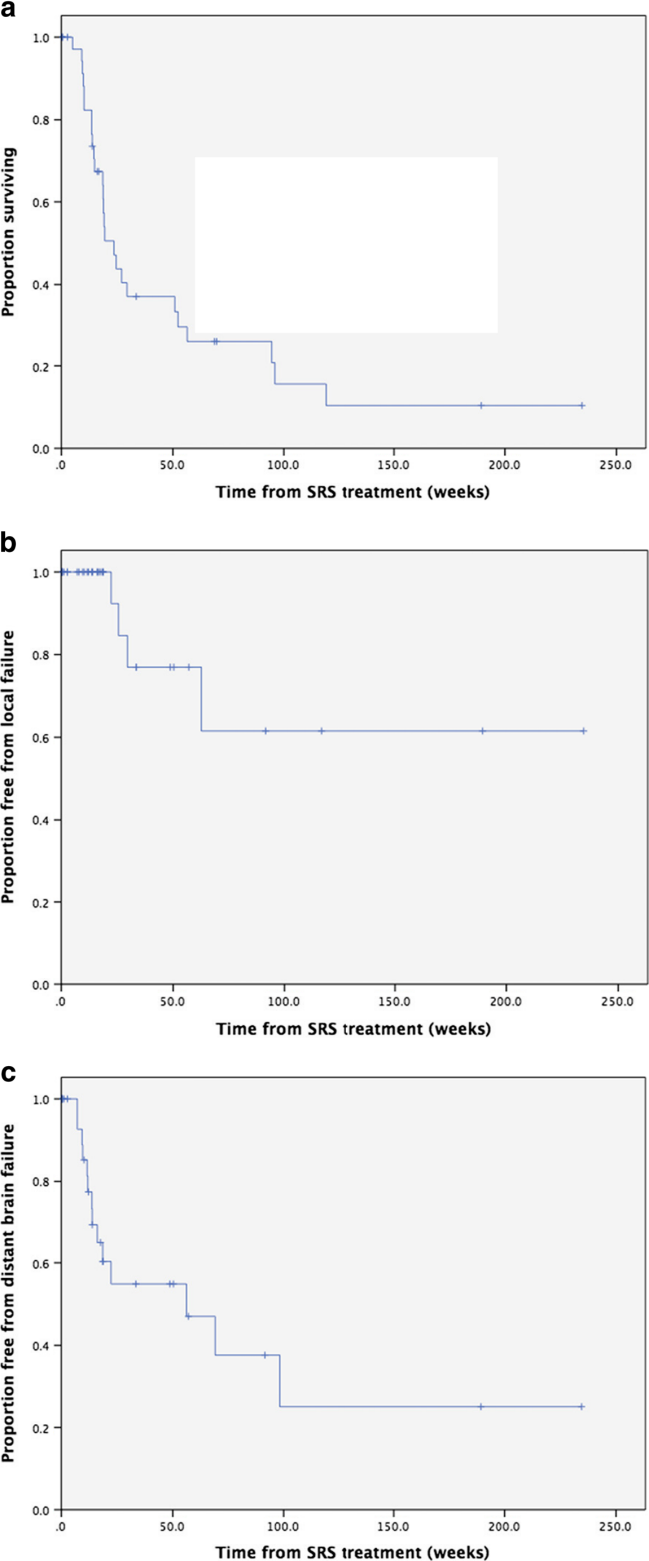
**a** Overall survival. **b** Freedom from local failure. **c** Freedom from distant brain failure

Median overall survival was 23.6 weeks (95 % CI: 16.2 to 31.0 weeks) with 1- and 2-year overall survival of 33.4 and 15.6 %, respectively (Fig. 1a). There were no deaths within 30 days of brainstem SRS.

Overall, 27 brainstem metastases (in 25 patients) were evaluable on follow-up MRI imaging. Response was unknown for the remaining 21 lesions (19 patients), due to death prior to the 3-month post-SRS scan (*n* = 9) or loss to imaging follow-up. Of the 27 lesions with imaging follow-up, there were 2 complete responses, 12 partial responses and 9 cases of stable disease. Local failure was recorded on 4 occasions in 3 patients, including the 1 patient who was treated with salvage radiosurgery to the same brainstem metastasis. The 6-month, 1- and 2-year freedom from local failure was 84.6, 76.9 and 61.5 %, respectively (Fig. 1b). Of the 3 patients who developed local failure, two had a diagnosis of non-small cell lung cancer and one had breast cancer. All 3 patients had received WBRT prior to brainstem SRS. In all 4 cases of local failure, the metastases were relatively small (volumes: 0.4–3.57 cc, maximum diameters: 10.5–21 mm). In all 3 patients with local failure, the marginal prescription dose was 15 Gy for the first brainstem SRS treatment. For the one patient (with a diagnosis of breast cancer) who received a second SRS treatment for local progression of the brainstem metastasis, a marginal dose of 12 Gy was prescribed for re-treatment. Following the brainstem SRS retreatment, there was radiological evidence of progression of the retreated brainstem lesion. This was accompanied by progression of additional non-brainstem brain metastases. Progression of all intra-cranial lesions continued on serial imaging, and so appearances were in keeping with disease progression and not brainstem radionecrosis. Thus, despite two SRS treatments to the same area (and previous WBRT), this patient did not develop radionecrosis or any other significant treatment-related toxicity.

Distant brain progression was reported in 14 patients of whom 8 (57.1 %) had received prior WBRT. Of the 29 patients who received WBRT prior to or concurrent with brainstem SRS, 8 (27.6 %) developed distant brain progression compared to 6 of the 12 patients (50 %) who received SRS alone. Within the whole group, median time to distant brain progression was 56.1 weeks with 6-month, 1- and 2-year freedom from distant brain progression of 54.8, 54.8 and 25.1 %, respectively (Fig. 1c).

### Treatment-related toxicity

Radionecrosis was observed in 4 patients (9 %), 2 of whom were symptomatic (4.5 % of all patients). Of the two symptomatic patients, one developed quadriparesis 6 months after SRS and improved clinically and on imaging with dexamethasone. The other symptomatic patient developed right leg pain, dizziness and speech difficulties 7 months after SRS. The patient had partial improvement with dexamethasone and subsequently received hyperbaric oxygen, which stabilised symptoms and improved the radiologically evident edema. The two asymptomatic patients developed radionecrosis 6 and 8 months following SRS. All 4 patients were prescribed marginal prescription doses of 15 Gy to relatively small brainstem lesions (target volumes: 1.26 cc to 3.18 cc, maximum dimensions: 19.0 to 22.4 mm) and all had received prior whole brain radiotherapy (20 Gy in 5 fractions in 2 patients and 30 Gy in 10 fractions in 2 patients). Other than these cases of radionecrosis, there were no other grade 3 or greater toxicities.

### Factors predictive of survival

**Fig. 2:**
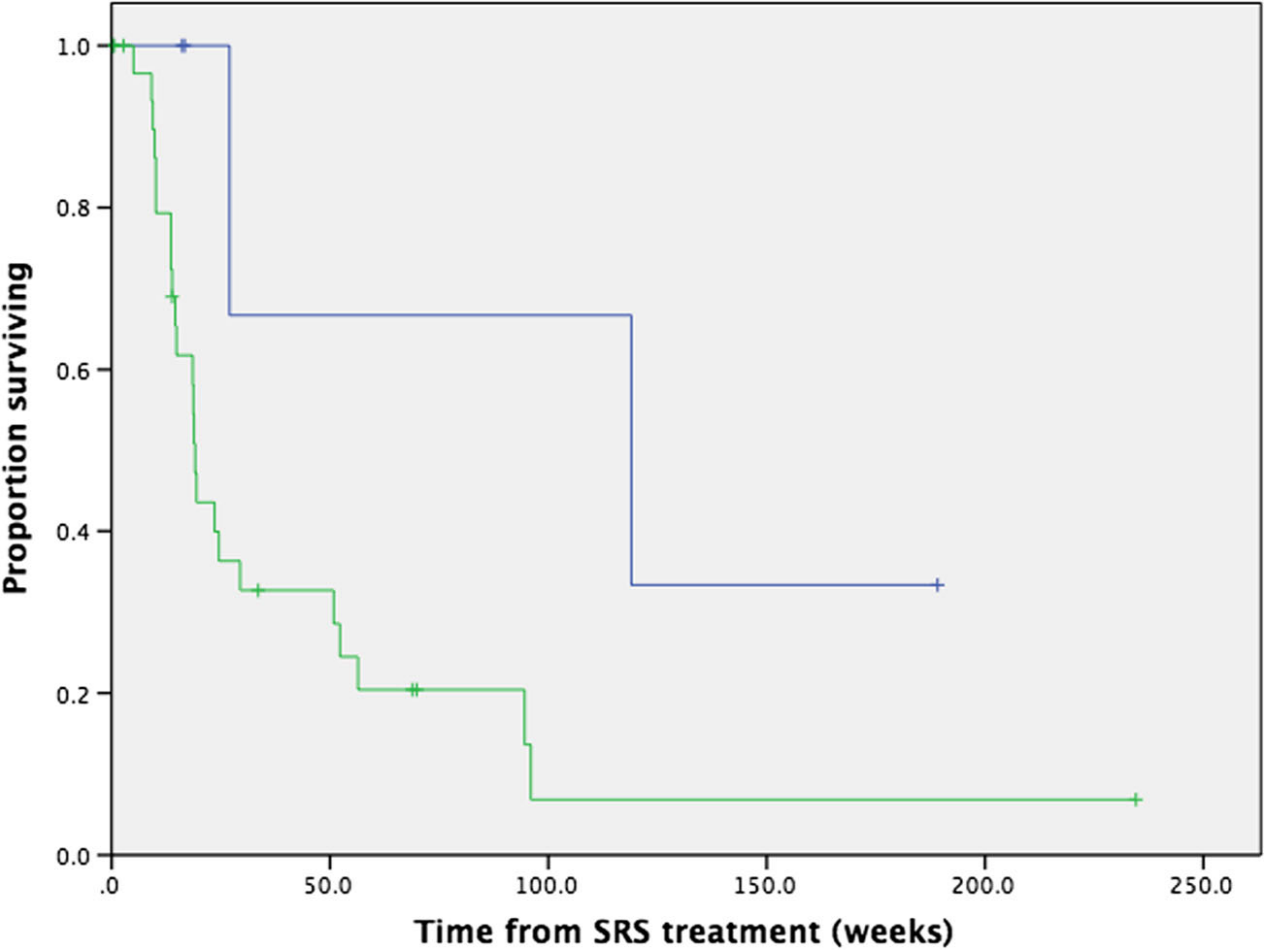
Patients receiving SRS for brainstem metastases, with or without additional external beam brain radiotherapy (*blue line*: no other brain radiotherapy, *green line*: other brain radiotherapy; *p* = 0.078)

On univariate analysis, no factors were identified that had a statistically significant impact on overall survival (Table 3). There was a trend towards better overall survival in patients who did not receive external beam brain radiotherapy (*p* = 0.078), which included WBRT (*n* = 33), base of skull (*n* = 2) or temporal bone (*n* = 1) radiotherapy (Fig. 2). Neither local failure, nor distant brain failure, had a statistically significant impact on overall survival. As above, given the small sample size and low number of events, statistical exploration for factors predictive of local control or toxicity was not performed.

**Table 3:** Univariate analysis for overall survival (hazard ratio and 95 % confidence interval only reported where *p* < 0.1)

Factor	*p* value	Hazard ratio	95 % confidence interval
Age	0.463		
Gender	0.745		
Additional external beam brain radiotherapy (received vs. not received)^a^	0.078	3.804	0.861–16.806
Synchronous non-brainstem brain metastases at time of brainstem SRS (present vs. absent)	0.250		
Histology of primary lesion	0.365		
Extra-cranial disease control at time of brainstem SRS	0.609		
Local brain failure (yes vs. no)	0.187		
Distant brain failure (yes vs. no)	0.592		

^a^Factor entered into multivariate model

## Discussion

Despite the precision and dose conformality of radiosurgery, there have been concerns about the safety of using SRS for the treatment of brainstem metastases. Our retrospective study supports that SRS for brainstem metastases results in good local control (76.9 % at 1 year) and is well tolerated by the majority of patients with symptomatic radionecrosis in only 2 patients (4.5 %). This is one of the largest series describing outcomes following WBRT in combination with brainstem SRS (*n* = 33), and to our knowledge, this is the largest reported experience of patients treated with a second course of WBRT at progression after brainstem SRS (*n* = 8). Our experience, along with 2 other studies involving a total of only 6 patients, demonstrates the safe delivery of 2 courses of WBRT with SRS to the brainstem without significant toxicity [9, 10].

In our review of the literature, a further 20 studies have specifically examined SRS for brainstem metastases using Gamma Knife or linear accelerator-based SRS (Table 4), [11–23, 10, 24, 9, 25–28]. Although patient numbers tend to be small, collectively, these studies report outcomes for over 1000 patients and over 1100 brainstem metastases treated with SRS. Based on these publications, we can generally conclude that high rates of local control can be achieved with infrequent serious toxicity in this patient population with relatively short overall survival. Median overall survival was 5.4 months in this current study, towards the lower end of that reported in other series with median survival ranging from 4.9 to 12 months (Table 4), perhaps reflecting the fact that most of the patients (75 %) in this series received brainstem SRS as a salvage treatment following previous WBRT, rather than as an upfront treatment option. It has been observed that brainstem metastases, more than other intra-cranial locations, have a negative impact on overall survival [29, 30], with median survivals of 4.4 and 6.5 months with and without brainstem metastases reported in one matched analysis [30]. Although the brainstem location appears to impact overall survival, neither local nor distant brain failure significantly impacted overall survival in our study.

**Table 4:** Studies investigating SRS for brainstem metastases

Study	Modality	Number of patients	Number of brainstem metastases	FU (months)	Volume of metastases (cc)	Marginal dose (Gy)	Local control (%)	OS (months)	WBRT (%; dosimetric details where available)	Toxicity
Huang et al., 1999^10^	GK	26	27	Median 9.5	Mean 2.0	Median 16.0	Crude 95 %	Median 9	92.3 % (median dose 30 Gy, range 23.4–44 Gy), 5 received extra plus 10 Gy at time of SRS as >3 months from WBRT	4 cases of mild and transient toxicity, 3 cases of seizures
Shuto et al., 2003^11^	GK	25	31	Mean 5.2	Mean 2.1	Mean 13.0	Crude 77.4 %	Median 4.9	28.0 % (range 30–45 Gy)	2 cases of brain injury
Fuentes et al. , 2006^12^	GK	28	NR	Median 12.5	Mean 2.1	Mean 16.6 if previous WBRT, Mean 20 Gy if no previous WBRT	Crude 92 %	Median 12	21.4 %	None
Hussain et al. 2007^13^	GK	22	25	Median 26.6	Median 0.9	Median 16	Crude 100 %	Median 8.5	13.6 %	1 episode of persistent hemiparesis
Yen et al., 2007^14^	GK	53	53	Mean 9.8	Mean 2.8	Mean 17.6		Median 11	39.6 %	None
Kased et al., 2008^15^	GK	42	44	Mean 6.9	Median 0.26	Median 16	1 year 77 %	Median 9	57.1 %	4 episodes of complications including 2 episodes of radionecrosis
Lorenzoni et al., 2009^24^	GK	25	27	Mean 10.5	Mean 0.6	Mean 20 Gy overall Mean 18.4 if previous brain RT, Mean 21.3 if no previous RT	2 years 89 %	Median 11.1	68.0 %	None
Samblas et al., 2009^16^	Linac	28	30	NR	Mean 1.86	Mean 11.1	Crude 95 %	Mean 16.8	96.4 % (30 Gy in 10 fractions)	None
Koyfman et al., 2010^17^	GK	43	43	Mean 5.3	Mean 0.37	Median 15.0	1 year 85 %	Median 5.8	79.1 % (median dose 35 Gy)	No grade 3+ toxicities, 3 episodes of grade 1 or 2 complications, 2 cases of radionecrosis on imaging
Hatiboglu et al., 2011^26^	Linac	60	60	Median 12.8	Median 1.0	Median 15	Crude 76 %	Median 4.0	25 %	12 episodes of complications (all grades), 2 major
Kelly et al., 2011^18^	Linac	24	NR	Median 6.6	Median 0.2	Median 13	1 year 78.6 %	Median 5.3	95.8 % (median dose 35 Gy, range 30–40 Gy)	2 episodes of grade 3 toxicity
Valery et al., 2011^25^	Linac	30	43	Mean 10.2	Mean 2.8	Mean 13.4	1 year 79 %	Median 10	26.7 %	No grade 3+ toxicity, 4 episodes of grade 1 or 2 complications
Yoo et al., 2011^19^	GK	32	32	Mean 12	Mean 1.5	Mean 15.9	Crude 87.5 %	Mean 7.7	NR	1 episode of haemorrhage
Kawabe et al., 2012^20^	GK	200	222	Median 5.8	Median 0.2	Median 18	2 year: 81.8 %	Median 6	6.5 %	1 episodes of severe oedema, 6 asymptomatic cases of oedema
Lin et al., 2012^21^	Linac	45	48	-	Median 0.40	Median 14	1 year 88 %	Median 11.6	46.7 % (median dose 37.5 Gy)	2 episodes of toxicity, rate 4.7 % at 2 years, 1 episode of symptomatic radionecrosis
Jung et al., 2013^22^	GK	32	32	Median 12.5	Median 0.71	Median 13	Crude 87.5 %	Median 5.2	53.1 % (30 Gy in 10 fractions)	None
Sengoz et al., 2013^9^	GK	44	46	NR	Median 0.6	Median 16	Crude 96 %	Median 8	65.9 %	2 episodes of asymptomatic peri-tumoral change on MRI
Kilburn et al., 2014^23^	GK	44	52	Median 6	Median 0.134	Median 18	1 year 74 %	Median 6	56.8 %	4 episodes of toxicity (3 grade 3), including 2 cases of suspected radionecrosis
Trifiletti et al., 2015	GK	161	189	Median 5.5	Median 0.4	Median 18	1 year 84.9 %	Median 5.5	51.6 %	3 episodes of severe toxicity including 1 possible episode of radionecrosis causing 2 severe toxicities
Voong et al., 2015^8^	GK	74	77	Median 5.5	Median 0.13	Median 16	1 year 85 %	Median 8.5	58.1 %	6 cases, including 2 cases of radionecrosis
Current study	GK	44	48	Median 16.1	Median 1.33	Median 15	1 year 79 %	Median 5.6	75.0 % (median 20 Gy, range 20–30 Gy)	4 cases of which 2 symptomatic

*NR* not reported, *WBRT* whole brain radiotherapy

There have been mixed reports about the impact of marginal dose on outcomes including overall survival and local control. Lorenzoni et al. identified that doses above 18 Gy predicted improved overall survival, and Trifiletti et al. and Kased et al. both demonstrated that doses of 16 Gy and above resulted in improved local control [16, 25, 28]. Other studies did not find marginal dose as an important predictor for local control or overall survival in patients treated with SRS for brainstem metastases [18, 19, 21, 24, 26]. Despite the majority of our patients receiving a lower marginal prescription dose of 15 Gy compared with other studies, local control was 78.6 % at 1 year in our series compared with at least 74 to 88 % in other series. (Table 4) and our overall survival was comparable to other studies. Relative to other studies that reported WBRT doses up to 45 Gy combined with SRS to the brainstem, the WBRT doses employed in our study were lower and hypofractionated [11, 12, 17–19, 22, 23], but outcomes were similar to published series. The impact of WBRT dose on local control and toxicity when used in conjunction with brainstem SRS has not been investigated or reported previously.

In this current study, brainstem SRS was well tolerated by most patients with 4 cases of radionecrosis as the only treatment-related toxicities, of which only 2 patients were symptomatic. In existing studies, serious toxicities have also been uncommon, consistently involving less than 10 % of patients (Table 4). Kilburn et al. observed that larger treatment volumes were predictive of toxicity in a series of 44 patients in whom 4 patients developed toxicity (one grade 2, three grade 3), including 2 patients who developed symptomatic radionecrosis (4.4 %) [24]. Our rate of symptomatic radionecrosis was similar at 4.5 %, but the target volumes in the cases that developed radionecrosis were small.

Due to the retrospective nature of this study, there are limitations with the data presented. Firstly, a relatively high proportion of patients were not evaluable for imaging response to brainstem SRS with about half of patients, who died prior to imaging and the other half who did not complete their 3-month post-treatment MRI. The large number lost to follow-up may reflect a combination of factors including the clinical state of these patients with advanced disease who may have been too unwell to attend for further imaging and the long distances patients have needed to travel for follow-up at this large tertiary cancer centre that received referrals from both nearby and remote regions. In addition, due to the retrospective nature of this study, we had incomplete data regarding the details of the presenting symptoms, consistent evaluation in any change of symptoms or dexamethasone use. Therefore, these variables were not included in any statistical analyses.

Despite the large collective number of patients now reported to have received SRS for brainstem metastases, as with this current series, individual studies tend to contain small numbers of patients, limiting the ability to identify factors predictive of local control or overall survival [19, 24, 26]. Even where statistically significant observations are made, one should exercise caution in the interpretation due to the small sample sizes relative to the number of predictive variables investigated. In this current study, trends were observed towards improved overall survival with the absence of additional fractionated brain radiotherapy and the absence of synchronous non-brainstem brain metastases at the time of SRS. In terms of the use of whole brain radiotherapy, Lorenzoni et al. and Jung et al. similarly found that the absence of whole brain radiotherapy was a significant predictor of improved survival [23, 25]. In both of these studies, like our study, the majority of patients received SRS as salvage treatment following initial whole brain radiotherapy. As such, these patients were likely later in their course of disease and therefore had an expected shorter survival following SRS than patients who had not received prior brain radiotherapy.

## Conclusion

This retrospective series of patients treated with SRS for brainstem metastases, largely in combination with at least one course of whole brain radiotherapy, demonstrates that this treatment approach is safe and results in good local control. Distant brain failure was lower in patients who received whole brain radiotherapy as part of initial brain metastasis treatment, as demonstrated in prior studies. In this cohort, no variables significantly impacted overall survival, including intracranial control.

## Electronic supplementary material

ESM 1 (PDF 38 kb)

## References

[1] Patchell RA (1991). Brain metastases. Neurol Clin.

[2] Cairncross JG, Kim JH, Posner JB (1980). Radiation therapy for brain metastases. Ann Neurol.

[3] Norden AD, Wen PY, Kesari S (2005). Brain metastases. Curr Opin Neurol.

[4] Lassman AB, DeAngelis LM (2003). Brain metastases. Neurol Clin.

[5] Delattre JY, Krol G, Thaler HT, Posner JB (1988). Distribution of brain metastases. Arch Neurol.

[6] Nieder C, Grosu AL, Gaspar LE (2014). Stereotactic radiosurgery (SRS) for brain metastases: a systematic review. Radiat Oncol.

[7] Therasse P, Arbuck SG, Eisenhauer EA, Wanders J, Kaplan RS, Rubinstein L, Verweij J, Van Glabbeke M, van Oosterom AT, Christian MC, Gwyther SG (2000). New guidelines to evaluate the response to treatment in solid tumors. European Organization for Research and Treatment of Cancer, National Cancer Institute of the United States, National Cancer Institute of Canada. J Natl Cancer Inst.

[8] Lin NU, Lee EQ, Aoyama H, Barani IJ, Barboriak DP, Baumert BG, Bendszus M, Brown PD, Camidge DR, Chang SM, Dancey J, de Vries EG, Gaspar LE, Harris GJ, Hodi FS, Kalkanis SN, Linskey ME, Macdonald DR, Margolin K, Mehta MP, Schiff D, Soffietti R, Suh JH, van den Bent MJ, Vogelbaum MA, Wen PY, Response Assessment in Neuro-Oncology group (2015). Response assessment criteria for brain metastases: proposal from the RANO group. Lancet Oncol.

[9] Voong KR, Farnia B, Wang Q, Luo D, McAleer MF, Rao G, Guha-Thakurta N, Likhacheva A, Ghia AJ, Brown PD, Li J (2015). Gamma knife stereotactic radiosurgery in the treatment of brainstem metastases: the MD Anderson experience. Neurooncol Pract.

[10] Sengoz M, Kabalay IA, Tezcanli E, Peker S, Pamir N (2013). Treatment of brainstem metastases with gamma-knife radiosurgery. J Neuro-Oncol.

[11] Huang CF, Kondziolka D, Flickinger JC, Lunsford LD (1999). Stereotactic radiosurgery for brainstem metastases. J Neurosurg.

[12] Shuto T, Fujino H, Asada H, Inomori S, Nagano H (2003). Gamma knife radiosurgery for metastatic tumours in the brain stem. Acta Neurochir.

[13] Fuentes S, Delsanti C, Metellus P, Peragut JC, Grisoli F, Regis J (2006). Brainstem metastases: management using gamma knife radiosurgery. Neurosurgery.

[14] Hussain A, Brown PD, Stafford SL, Pollock BE (2007). Stereotactic radiosurgery for brainstem metastases: survival, tumor control, and patient outcomes. Int J Radiat Oncol Biol Phys.

[15] Yen CP, Sheehan J, Patterson G, Steiner L (2006). Gamma knife surgery for metastatic brainstem tumors. J Neurosurg.

[16] Kased N, Huang K, Nakamura JL, Sahgal A, Larson DA, McDermott MW, Sneed PK (2008). Gamma knife radiosurgery for brainstem metastases: the UCSF experience. J Neuro-Oncol.

[17] Samblas JM, Sallabanda K, Bustos JC, Gutierrez-Diaz JA, Peraza C, Beltran C, Samper PM (2009). Radiosurgery and whole brain therapy in the treatment of brainstem metastases. Clin Transl Oncol.

[18] Koyfman SA, Tendulkar RD, Chao ST, Vogelbaum MA, Barnett GH, Angelov L, Weil RJ, Neyman G, Reddy CA, Suh JH (2010). Stereotactic radiosurgery for single brainstem metastases: the Cleveland clinic experience. Int J Radiat Oncol Biol Phys.

[19] Kelly PJ, Lin YB, Yu AY, Ropper AE, Nguyen PL, Marcus KJ, Hacker FL, Weiss SE (2011). Linear accelerator-based stereotactic radiosurgery for brainstem metastases: the Dana-Farber/Brigham and Women’s Cancer Center experience. J Neuro-Oncol.

[20] Yoo TW, Park ES, Kwon do H, Kim CJ (2011). Gamma knife radiosurgery for brainstem metastasis. J Korean Neurosurg Soc.

[21] Kawabe T, Yamamoto M, Sato Y, Barfod BE, Urakawa Y, Kasuya H, Mineura K (2012). Gamma Knife surgery for patients with brainstem metastases. J Neurosurg.

[22] Lin CS, Selch MT, Lee SP, Wu JK, Xiao F, Hong DS, Chen CH, Hussain A, Lee PP, De Salles AA (2012). Accelerator-based stereotactic radiosurgery for brainstem metastases. Neurosurgery.

[23] Jung EW, Rakowski JT, Delly F, Jagannathan J, Konski AA, Guthikonda M, Kim H, Mittal S (2013). Gamma Knife radiosurgery in the management of brainstem metastases. Clin Neurol Neurosurg.

[24] Kilburn JM, Ellis TL, Lovato JF, Urbanic JJ, Bourland JD, Munley MT, Deguzman AF, McMullen KP, Shaw EG, Tatter SB, Chan MD (2014). Local control and toxicity outcomes in brainstem metastases treated with single fraction radiosurgery: is there a volume threshold for toxicity?. J Neuro-Oncol.

[25] Lorenzoni JG, Devriendt D, Massager N, Desmedt F, Simon S, Van Houtte P, Brotchi J, Levivier M (2009). Brain stem metastases treated with radiosurgery: prognostic factors of survival and life expectancy estimation. Surg Neurol.

[26] Valery CA, Boskos C, Boisserie G, Lamproglou I, Cornu P, Mazeron JJ, Simon JM (2011). Minimized doses for linear accelerator radiosurgery of brainstem metastasis. Int J Radiat Oncol Biol Phys.

[27] Hatiboglu MA, Chang EL, Suki D, Sawaya R, Wildrick DM, Weinberg JS (2011). Outcomes and prognostic factors for patients with brainstem metastases undergoing stereotactic radiosurgery. Neurosurgery.

[28] Trifiletti DM, Lee CC, Winardi W, Patel NV, Yen CP, Larner JM, Sheehan JP (2015). Brainstem metastases treated with stereotactic radiosurgery: safety, efficacy, and dose response. J Neuro-Oncol.

[29] Lucas JT, Colmer HG, White L, Fitzgerald N, Isom S, Bourland JD, Laxton AW, Tatter SB, Chan MD (2015). Competing risk analysis of neurologic versus nonneurologic death in patients undergoing radiosurgical salvage after whole-brain radiation therapy failure: who actually dies of their brain metastases?. Int J Radiat Oncol Biol Phys.

[30] Trifletti DM, Lee CC, Shah N, Patel NV, Chen SC, Sheehan JP (2015). How does brainstem involvement affect prognosis in patients with limited brain metastases? Results of a matched-cohort analysis. World Neurosurgery.

