# Supplementary material for: Radiosurgery for brainstem metastases with and without whole brain radiotherapy: clinical series and literature review
Source: J Radiat Oncol. 2016 Oct 27;6(1):21–30. doi: 10.1007/s13566-016-0281-4 (PMC5357261; doi:10.1007/s13566-016-0281-4)
Supplement: Supplementary file 1 — (PDF 38 kb) [file 13566_2016_281_MOESM1_ESM.pdf]

Electronic Supplementary Material:

Clinical Outcomes of Radiosurgery for Brainstem Metastases

Louise Murray [1], Cynthia Menard[1], Gelareh Zadeh[2], Karolyn Au[2], Mark Bernstein[2], Barbara-Ann Millar [1], Normand Laperriere[1], Caroline Chung[1].

1. Department of Radiation Oncology, University of Toronto, Princess Margaret Cancer Centre, Toronto, Ontario, Canada
2. Division of Neurosurgery, University of Toronto, Toronto Western Hospital, Toronto, Ontario, Canada

Short title: SRS for brainstem metastases

Corresponding author:
Caroline Chung MD FRCPC CIP
Radiation Medicine Program,
Princess Margaret Cancer Centre,
610 University Avenue,
Toronto
Canada M5G 2M9
E-mail: caroline.chung.md@gmail.com, cchung3@mdanderson.org

Online Resource 1

Whole brain radiotherapy before and after brainstem stereotactic radiosurgery

Initial WBRT dose and fractionation	Repeat WBRT dose and fractionation	Number of patients	Combined EQD2 to normal brain tissue (α/β= 2 Gy for late effects)
20 Gy, 5 fractions	25 Gy, 10 fractions	2	58.1 Gy
20 Gy, 5 fractions	20 Gy, 10 fractions	2	50 Gy
30 Gy, 10 fractions	25 Gy, 10 fractions	2	65.6 Gy
30 Gy, 10 fractions	20 Gy, 10 fractions	1	57.5 Gy
20 Gy, 5 fractions	15 Gy, 5 fractions	1	48.8 Gy
